# Survey of Pulse Crop Field for Plant-Parasitic Nematodes in the Canadian Prairies

**DOI:** 10.2478/jofnem-2025-0040

**Published:** 2025-12-31

**Authors:** F. Gouvea-Pereira, M. Tenuta, D. Risula, M. W. Harding

**Affiliations:** Department of Soil Science, University of Manitoba, Winnipeg, MB, R3T 2N2, Canada; Government of Saskatchewan. Special Crops. 125 - 3085 Albert Street, Regina, SK, S4S 0B1, Canada; Alberta Agriculture and Irrigation, Crop Diversification Center South, 301 Horticultural Station Road East, Brooks, AB T1R 1E6, Canada

**Keywords:** Alberta, Canadian Prairies, chickpea, creeping thistle, detection, diagnosis, *Ditylenchus dipsaci*, *Helicotylenchus*, host-parasitic relationship, lentil, Manitoba, molecular biology, *Paratylenchus projectus*, pea, pin nematode, *Pratylenchus neglectus*, pulse, *Quinisulcius capitatus*, regulatory, root-lesion nematode, Saskatchewan, stem and bulb nematode, survey, Telotylenchinae, yellow pea

## Abstract

The distribution of economically significant plant-parasitic nematodes in pulse crops in the Canadian Prairies is relatively unknown. Reports suggested that *Ditylenchus dipsaci* in yellow pea export was likely the nonquarantine species *D. weischeri*, a Canada thistle (*Cirsium arvense*) parasite. To determine if *D. dipsaci* is found in pulse plants and understand nematode distribution in the Canadian Prairies, a survey was conducted in commercial yellow pea, lentil and chickpea fields in Alberta, Saskatchewan, and Manitoba. Samples of pulse and thistle plants (flowers or pods, stems and leaves) and soil were collected from 94 fields. Nematodes were identified by morphological features and molecular analyses (species-specific PCR, PCR-RFLP, and sequencing of the partial 18S, 28S and ITS of the rDNA gene). High densities of plant-parasitic nematodes — *Pratylenchus, Paratylenchus, Helicotylenchus* and Telotylenchinae — were found in several fields. *Ditylenchus weischeri*, a parasite of thistles and not pulse crops, was recovered from 20 fields across Alberta, Saskatchewan and Manitoba; *D. dipsaci* was found in pods of one yellow pea field in Manitoba. These results confirm the high prevalence of *D. weischeri* on creeping thistle in pulse fields and the near absence of the quarantine pest *D. dipsaci*.

Canada is the world's largest exporter of pulses; more than 85% of its production is exported to more than 120 countries (Pulse Canada, 2024). The main pulses grown in Canada are dry peas (*Pisum sativum* L.), lentils (*Lens culinaris* L.), chickpeas (*Cicer arietinum* L.) and dry beans (*Phaseolus vulgaris* L.) (Pulse Canada, 2024).

The largest pulse-growing areas are situated in the Canadian Prairies due to favorable agricultural conditions, such as suitable climate and fertile soil (Bekkering, 2015). Saskatchewan and Alberta account for most of the pulse-producing area, with the remaining area in Manitoba (Bekkering, 2015).

Some plant-parasitic nematodes can negatively impact international markets' access, as is the case with the quarantine pest *Ditylenchus dipsaci* (Kühn, 1857) Filipjev, 1936, which has been particularly problematic for yellow pea exports from Canada to India. *Ditylenchus dipsaci* is a crop pest of quarantine status in many countries due to its wide host range and ability to cause extensive economic losses (Bélair et al., 2018). Watson and Shorthouse (1979) reported *D. dipsaci* infesting Canada thistle (*Cirsium arvense* L.) in Saskatchewan. More recently, a new *Ditylenchus* species, *D. weischeri* Chizhov, Borisov and Subbotin, 2010, was described parasitizing thistle in Russia (Chizhov et al., 2010). Following this new finding, Tenuta et al. (2014) conducted studies in the Prairie provinces to determine the phytosanitary risks of pea grain exports containing *D. dipsaci*. The results indicated that *D. weischeri*, but probably not *D. dipsaci*, was present in 2009 and 2010 yellow pea grain harvest samples and Canada thistle plants in Alberta, Saskatchewan, and Manitoba (Tenuta et al., 2014). *Ditylenchus weischeri* parasitizes Canada thistle and is not an agricultural pest of crops grown in the Canadian Prairies (Hajihassani et al., 2016, 2017).

Current knowledge of the biodiversity of plant-parasitic nematodes of crops in Canada is predominantly for cropped regions of British Columbia, Ontario, Quebec, New Brunswick, Nova Scotia, and Prince Edward Island (Kimpinski and Thompson 1990; Potter and McKeown 2003). Few recent surveys have been conducted in the Prairie provinces (Webster and Hawn 1973; Hawn 1973; Sewell 1977; Ebsary et al. 1984; Vrain and Ebsary 1994; Potter and McKeown 2003; Holzgang and Pearse 2006; Forge et al. 2019). Data on plant-parasitic nematodes associated with pulse crops in Canada are limited and are limited to surveys and research conducted several decades ago. The specimens described also may now be subject to a reinterpretation of identity due to recent developments in molecular identification methods (Potter and McKeown, 2003) and changes in nematode distribution and population densities.

The impact of plant-parasitic nematodes on pulse crop yield in the Canadian Prairies remains largely unexplored. However, in other pulse growing regions, several pulse crops are significantly affected by nematodes. For example, peas are affected by *D. dipsaci*; *Pratylenchus neglectus* (Rensch, 1924) Filipjev & Schuurmans Stekhoven, 1941; *Pratylenchus thornei* Sher & Allen, 1953; and *Paratylenchus hamatus* Thorne & Allen, 1950. Lentils are affected by *D. dipsaci; Pratylenchus lentis* Troccoli, 2008; *P. neglectus; P. thornei;* and *P. hamatus* (Hafez et al., 2010). Chickpeas are affected by *D. dipsaci, P. neglectus*, and *P. thornei*. Faba beans are subject to yield loss due to *Ditylenchus gigas* Vovlas, Troccoli, Palomares-Rius, De Luca, Liebanas, Landa, and Subbotin & Castillo, 2011 (Singh et al., 2013). Significant crop losses of up to 90% due to these nematodes have been reported, for example, in Idaho in the United States, in lentil and pea fields infested with *P. neglectus*, *P. thornei*, and *P. hamatus* (Riga et al., 2008).

The prevalence of plant-parasitic nematodes in pulse crops in Western Canada is relatively unknown. This research aims to address this knowledge gap and investigate the distribution of the quarantine pest *D. dipsaci* as well as other agriculturally significant plant-parasitic nematodes in pulse crops on the Canadian Prairies.

## Materials and Methods

**Figure 1:**
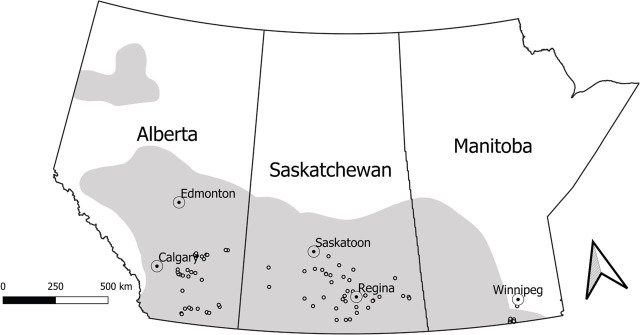
Pulse growing areas in the Canadian Prairie provinces (gray shading) and locations of commercial fields sampled in 2014 and 2015 (n = 94).

*Soil and plant sampling*: A total of 466 soil and plant samples of yellow pea, chickpea, lentil and Canada thistle from 94 commercial fields were surveyed for the identification of the occurrence of *Ditylenchus* and other plant-parasitic nematodes in Alberta, Saskatchewan, and Manitoba during the summers of 2014 and 2015 (Table 1, Fig. 1). Thistle plants were also examined to confirm infestation by *D. weischeri*. In each field, above-ground biomass was collected from five randomly-selected thistle plants and ten pulse crop plants using a “W” pattern walk. Plant samples were combined to make one crop and one thistle plant sample per field. A split-tube sampler (3.5-cm diam.) was used to collect one soil core (0–30 cm) from the base of each harvested crop, or two from each thistle plant. Each set of ten cores was pooled to make one crop and one thistle soil sample for each field. Samples were refrigerated and shipped to the University of Manitoba Applied Soil Ecology Laboratory in chest coolers.

**Table 1:** Number of fields surveyed and plant and soil samples collected from commercial farms in the Canadian Prairie provinces.

			**Sample Types**						
			**Crop**			**Canada Thistle**			**Total**
				
**Province**		**Fields**	**Pods**	**Stems/Leaves**	**Soil**	**Flowers**	**Stems/Leaves**	**Soil**	
Saskatchewan	Pea	31	31	31	31	25	31	31	180
Saskatchewan	Lentil	13	13	13	13	0	0	0	39
Saskatchewan	Chickpea	3	2	3	3	0	0	0	8
Alberta	Pea	33	25	32	33	17	27	28	162
Alberta	Chickpea	7	7	7	7	5	6	3	35
Manitoba	Pea	7	7	7	7	7	7	7	42

Total		94	85	93	94	54	71	69	466

Nematode extraction, morphological identification and enumeration: Nematode extraction was performed from both plant and soil samples. The total number of soil and plant sample types extracted for analysis is presented in Table 1.

Nematodes were extracted from plant materials using a modified Whitehead tray method (Whitehead and Hemming, 1965). Each plant sample was divided into two sub-samples based on plant parts: (1) stems and leaves and (2) flowers (for thistle) or pods and seeds (for pea, chickpea, and lentil). Sub-samples were chopped to a maximum length of 1 cm and weight of 5 g for stems and leaves, 5 g for thistle flowers, and 10 g for seeds, then placed in extraction units. Each unit consisted of a 18-cm-diam. nursery pot saucer lined with a 700-μm-mesh wire, supported by three 3-mm-thick plastic rings, and covered with single-ply paper (Kimwipe, Kimtech Science, Mississauga, ON, Canada) wetted with reverse osmosis water. Subsamples were placed on the paper and covered with a second overturned saucer. Extraction units were incubated in a dark controlled environment room at 21°C for either six days (stems and leaves) or four days (pods and flowers). Water was added as needed to keep plant material saturated. After incubation, the suspension from each saucer was emptied onto a stack of sieves (140-μm-mesh above 33-μm-mesh). Screens were rinsed several times with tap water, trapping nematodes on the second screen. Trapped nematodes and plant material were washed into a 15-ml conical centrifuge flask and stored at 4°C until analysis.

Nematodes in soil samples were extracted from a 100-g subsample using the sieving-sugar flotation method (Jenkins, 1964; Ingham, 1994). Gravimetric soil moisture content was determined for each soil sample. The entire extracted suspension was counted under an inverted microscope using a gridded dish at 40× magnification. After the total count, slides were prepared, and the first 100 nematodes were identified based on morphological characters using standard taxonomic keys (Mai et al., 1996; Mekete et al., 2012) under a bright-field microscope at magnifications of 400× and 1000×. Nematodes were identified to the genus or family level, and those lacking stylets were enumerated and categorized as non-plant-parasitic nematodes.

The total nematode count was adjusted based on the gravimetric soil moisture content and multiplied by the relative abundance (out of 100) of each taxon to estimate the actual abundance of nematodes for that taxon per 100 g of dry soil.

*Nematode molecular identification — DNA extraction*: DNA extraction followed a slightly modified version of the protocol described by Tenuta et al. (2014). A single nematode was hand-picked using a handling needle; transferred onto an embryo dish under a dissecting microscope; rinsed at least three times in sterile (autoclaved) ddH_2_O; transferred to a 0.2-ml PCR reaction tube containing 10 μl sterile ddH_2_O, 2 μl of Proteinase K (Roche, UK) and 12 μl of Direct PCR Lysis Reagent (Viagen Biotech, Los Angeles, CA); and frozen at −80°C overnight. The tube was then placed in a Thermocycler (T100TM, Bio-Rad Laboratories Canada Ltd, Mississauga, ON) machine, heated at 60°C for 60 minutes then at 94°C for 10 minutes. The DNA was stored at −20°C until PCR.

*Nematode molecular identification — polymerase chain reaction (PCR):* This study used 13 nematode-universal and species-specific PCR primer sets for DNA analyses (Table 2). The PCR mixture consisted of 1 to 6 μl of DNA extraction solution; 2.5 μl of 10× PCR buffer; 1 μl of dNTPs mixture (dATP, dCTP, dGTP and dTTP); 0.2 μl of DreamTaq DNA polymerase (Thermo Fisher Scientific, Waltham, MA); 250 nM of each primer set; and ddH_2_O, to a final volume of 25 μl. PCR amplification conditions for each primer set are given in Table 2. Amplification products were isolated by electrophoresis on 1.5% agarose gels with 1% TAE buffer, stained with 1 μl of 10,000× concentrated GelRed fluorescent dye (Biotium Inc, Hayward, CA), and visualized under UV illumination using a Gbox gel-capture imaging system (SYNGENE, Synoptic, Cambridge, UK). Positive controls were included for *Ditylenchus weischeri*, *D. dipsaci*, and *Pratylenchus neglectus*. For other species, including *P. penetrans*, *P. thornei*, *P. scribineri*, *A. besseyi*, *A. ritzemabosi*, *A. fragariae*, and *A. subtenuis*, positive controls were not available; consequently, we conducted sequencing for possible species identification.

**Table 2:** Primers used for PCR reactions and sequencing in this study.

**Primer Name**	**Specificity**	Product length (bp)^*^	**Target gene or fragment**	**Primer Sequence 5′-3′**	**Reference**
AB28-TW81	Universal	700–1100	ITS 1&2	ATA TGC TTA AGT TCA GCG GGT GGT TCC GTA GGT GAA CCT GC	Howlett et al. (1992) and Joyce et al. (1994)
D2A-D3B	Universal	800–1000	(D2–D3) 28S	ACA AGT ACC GTG AGG GAA AGT T TCG GAA GGA ACC AGC TAC TA	De Ley et al. (2005)
ITSF-ITSR	Universal	500–700	ITS 1	TTG ATT ACG TCC CTG CCC TTT ACG AGC CGA GTG ATC CAC CG	Vrain et al. (1992) and Cherry (1997)
18SF-18SR	Universal	900	18S	TTG GAT AAC TG TGG TTT AAC TAG ATT TCA CCT CTC ACG CAA CA	Qiao et al. (2016)
U831-Dipsaci_hsp90R	*D. dipsaci*	190	hsp90	AAY AAR ACM AAG CCN TYT GGA C GWG TTA WAT AAC TTG GTC RGC	Madani et al. (2015)
U831-Weischeri_hsp90R	*D. weischeri*	200	hsp 90	AAY AAR ACM AAG CCN TYT GGA C AGC ACT AAA ATT AAG YGT AAA GG	Madani et al. (2015)
PNEG-D3B	*P. neglectus*	290	(D3) 26S rDNA	ATG AAA GTG AAC ATG TCC TC TCG GAA GGA ACC AGC TAC TA	Al-Banna et al. (2004)
PPEN-D3B	*P. penetrans*	278	(D3) 26S rDNA	TAA AGA ATC CGC AAG GAT AC TCG GAA GGA ACC AGC TAC TA	Al-Banna et al. (2004)
PSCR-D3B	*P. scribneri*	286	(D3) 26S rDNA	AAA GTG AAC GTT TCC ATT TC TCG GAA GGA ACC AGC TAC TA	Al-Banna et al. (2004)
PTHO-D3B	*P. thornei*	288	(D3) 26S rDNA	GAA AGT GAA GGT ATC CCT CG TCG GAA GGA ACC AGC TAC TA	Al-Banna et al. (2004)
1770–1772	*A. besseyi*	325	SSU rDNA	GCG GGA TTC GTG GTT C*T CGA CAT GCC GAA ACA TGA G	Rybarczyk-Mydłowska et al. (2012)
1496–1499	*A. ritzemabosi*	347	SSU rDNA	CGC TGG TGG GTT TCG A CCC GCT AAG AAA TGA TCA C*C	Rybarczyk-Mydłowska et al. (2012)
AFragF1-AFragR1	*A. fragariae*	169	ITS1	GCA AGT GCT ATG CGA TCT TCT GCC ACA TCG GGT CAT TAT TT	McCuiston et al. (2007)

*Nematode molecular identification* — *sequencing*: Four sets of universal primers were selected to amplify regions of the 18S small subunit (SSU), 28S large ribosomal subunit (LSU), and internal transcribed spacer (ITS) of the rDNA gene (Table 2). The internal transcribed spacer regions ITS1 and ITS2 of the rDNA gene were amplified using the primers ITS1-F (Vrain et al., 1992) and ITS1-R (Cherry et al., 1997), as well as AB28-TW81 (Joyce et al., 1994). The large ribosomal subunit (LSU) D2-D3 expansion segment and the partial 18S region of the rDNA gene were amplified using the D2A-D3B primers (De Ley et al. 2005) and 18SF-18S-R (Qiao et al., 2016), respectively.

DNA fragments were purified either from agarose gels using either QIAquick Gel Extraction Kit (QIAGEN, Hilden, Germany) or QIAquick PCR Purification Kit (QIAGEN, Hilden, Germany), following the manufacturer's instructions. Amplification product concentration and quality were assessed using a fluorometer (Qubit, Thermo Fisher Scientific, Waltham, MA) and a spectrophotometer (NanoDrop 2000, Wilmington, DE), respectively. Sequencing for the obtained PCR amplicons was performed by Psomagen, Inc. (Rockville, MD). The sequencing data were analyzed using BLAST (Basic Local Alignment Search Tool) against the NCBI (National Center for Biotechnology Information) nucleotide database. Species-level assignment was based on the best match criteria, where an identity score of ≥ 98%, a query coverage percentage of ≥ 97%, and an expected value (E-value) of ≤ 0 were required for the sequences to be considered for species assignment.

Sequences targeting various regions from the nematodes obtained in this study were submitted to GenBank under the following accession numbers: *Ditylenchus weischeri* (OR636498, OR636499, OR644164, OR636500); *Pratylenchus neglectus* (OR644165, OR644166, OR636619); and *Paratylenchus projectus*, Jenkins, 1956 (OR644167, OR636501, OR636620).

*Restriction Fragment Length Polymorphism (PCR-RFLP):* Amplified rDNA-ITS products of *Ditylenchus* spp. were subjected to restriction fragment analysis as an additional species identification method. PCR-RFLP reactions were prepared using the same procedure as Tenuta et al. (2014).

*Data analyses*: Each nematode taxon's population density in soil was reported on a dry-weight soil basis (nematode number per 100 g^−1^ dry soil) and in plant components on a fresh-weight basis (nematode number per g^−1^). Chi-square tests were conducted for each nematode genus to assess differences in the proportion of positive samples between crop and thistle samples, with the null hypothesis stating that the prevalence of the nematode genus was equal in crop and thistle samples. Analyses were conducted separately for above-ground samples, which included *Aphelenchoides*, Aphelenchidae and *Ditylenchus*, and for soil samples, which included *Aphelenchoides*, Aphelenchidae, *Ditylenchus*, *Helicotylenchus*, *Paratylenchus*, *Pratylenchus*, and Telotylenchinae. All statistical analyses were carried out using SAS University Edition (SAS Institute, Cary, NC, USA), with a significance level set at 5%.

## Results

Sixty percent of all 466 samples analyzed had at least one nematode from a genus known to contain plant-parasitic nematodes. Twenty percent of the samples had only free-living nematodes. The other 20% had no nematodes at all; these samples were taken from above-ground plant parts.

Twenty-one genera containing plant-parasitic nematodes were recovered from the soil and / or plant-parts of peas, chickpeas, lentils and thistle plants from the Canadian Prairies, namely *Anguina*, *Aphelenchoides*, *Aphelenchus, Coslenchus, Ditylenchus*, *Filenchus, Helicotylenchus*, *Hoplolaimus*, *Longidorus*, *Merlinius*, *Paraphelenchus, Paratrichodorus, Paratylenchus*, *Pratylenchus*, *Psilenchus*, *Subanguina*, *Trichodorus*, *Tylenchorhynchus, Quinisulcius, Tylenchus* and *Xiphinema*.

*Aphelenchoides* (Aphelenchoididae) nematodes were the most widely found taxon (present in 76% of the surveyed fields), followed by Telotylenchinae (64%), *Aphelenchidae* (54%), *Ditylenchus* (52%)*, Paratylenchus* (49%), *Helicotylenchus* (21%), and *Pratylenchus* (20%) (Table 3). Chi-square analysis showed that *Ditylenchus* was found in a greater proportion of above-ground thistle samples than in above-ground crop samples (χ^2^ = 15.71, *P* < 0.0001). However, no significant difference in *Ditylenchus* prevalence was observed between soil samples from crops and samples from thistles (χ^2^ = 0.16, *P* = 0.68). *Aphelenchoides* were found more frequently in above-ground crop samples than in thistle samples (χ^2^ = 4.68, P = 0.03), and Aphelenchidae were found more frequently in above-ground crop (χ^2^ = 4.43, *P* = 0.03) and soil samples (χ^2^ = 4.25, *P* = 0.03) than in the same sample types from thistle plants. No other taxa differed in frequency of occurrence between the crop and thistle samples (Table 3).

**Table 3:** Proportion of fields and samples (above ground plant tissue and soil for crops and thistles) positive for taxa of plant-parasitic nematodes, and chi-square tests for comparison of crop to thistle samples, in commercial fields in the Canadian Prairies.

**Taxa**	**Fields**	**Sample**							
		**Above-Ground Crop**	**Above-Ground Thistle**	**χ^2^**	**p-value**	**Soil Under Crop**	**Soil Under Thistle**	**χ^2^**	**p-value**
							
		***proportion***				***proportion***			
*Anguina*	0.03	0.02	–^a^	–	–	–	–	–	–
*Aphelenchoides*	0.76	0.24	0.14	4.68	0.03^*^	0.46	0.48	0.07	0.79
Aphelenchidae	0.54	0.12	0.05	4.43	0.03^*^	0.35	0.20	4.25	0.03^*^
*Ditylenchus*	0.52	0.07	0.22	15.71	<.0001^*^	0.19	0.22	0.17	0.68
*Helicotylenchus*	0.21	–	–	–	–	0.16	0.19	0.23	0.63
*Hoplolaimus*	0.02	–	–	–	–	–	0.01	–	–
*Longidorus*	0.01	–	–	–	–	–	0.01	–	–
Merliniinae	0.01	–	–	–	–	0.01	–	–	–
*Paratrichodorus*	0.01	0.01	–	–	–	–	–	–	–
*Paratylenchus*	0.49	–	–	–	–	0.38	0.36	0.07	0.79
*Pratylenchus*	0.20	–	–	–	–	0.16	0.12	0.63	0.43
*Subanguina*	0.05	0.02	0.03	–	–	–	–	–	–
Telotylenchinae	0.64	0.01	–	–	–	0.47	0.50	0.43	0.62
*Xiphinema*	0.06	–	–	–	–	0.03	0.04	–	–

Number Examined	94	178	125			94	69		

a – = absence of nematode population of that taxon.

Asterisk (^*^) means a significant difference in the proportion of positive samples within above-ground samples or within soil samples for crops and thistles (p < 0.05).

*Nematode abundance in pea fields*: In pea fields, most nematode genera were found in above-ground plant samples at mean densities below eight nematodes per gram of plant tissue, with the exception of *Ditylenchus*, which had a mean density of 55 nematodes per g^−1^ in thistle flowers and a maximum density of 300 nematodes per g^−1^ (Table 4). The mean population density of *Ditylenchus* in thistle flowers was greater than in pea pods and pea stem and leaf samples (Table 4). Several nematode genera exhibited notable densities in soils from pea fields. The highest mean densities were observed for *Paratylenchus* (131 nematodes per 100 g^−1^ dry pea soil), *Helicotylenchus* (115 and 110 nematodes per 100 g^−1^ dry pea and thistle soil, respectively), *Pratylenchus* (106 nematodes per 100 g^−1^ dry pea soil), and Telotylenchinae (106 nematodes per 100 g^−1^ dry thistle soil) (Table 4).

**Table 4:** Mean population densities for plant samples (nematodes per gram) and soil (nematodes per 100 g dry soil mass) positive for taxa of plant-parasitic nematodes from commercial pea fields in the Canadian Prairies sampled in 2014 and 2015.

**Taxa**	**Above-Ground Crop**				**Above-Ground Thistle**							
	**Pods**		**Stems/Leaves**		**Flowers**		**Stems/Leaves**		**Soil Under Crop**		**Soil Under Thistle**	
	**Mean**	**Max^a^**	**Mean**	**Max**	**Mean**	**Max**	**Mean**	**Max**	**Mean**	**Max**	**Mean**	**Max**
*Anguina*	<1	n/a^b^	1	n/a	–^c^	–	–	–	–	–	–	–
*Aphelenchoides*	<1	1	5	25	<1	<1–1	<1	10	32	159	28	97
Aphelenchidae	<1	n/a	2	12	–	–	1	3	22	79	31	91
*Ditylenchus*	1	2	4	14	55	<1–300	27	37	28	91	82	332
*Helicotylenchus*	–	–	–	–	–	–	–	–	115	506	110	328
*Hoplolaimus*	–	–	–	–	–	–	–	–	–	–	2	n/a
*Longidorus*	–	–	–	–	–	–	–	–	–	–	7	n/a
Merliniinae	–	–	–	–	–	–	–	–	10	n/a	–	–
*Paratrichodorus*	–	–	8	n/a	–	–	–	–	–	–	–	–
*Paratylenchus*	–	–	–	–	–	–	–	–	131	1024	66	420
*Pratylenchus*	–	–	–	–	–	–	–	–	106	630	87	176
*Subanguina*	<1	n/a	6	11	–	–	1	1	8	n/a	–	–
Telotylenchinae	–	–	1	n/a	–	–	–	–	74	659	106	980
*Xiphinema*	–	–	–	–	–	–	–	–	13	18	10	15

a Max = highest population density observed in a sample.

b n/a = not provided because nematode genera was found in a single sample.

c – = absence of nematode population of that taxon.

Overall, both pea crops and Saskatchewan fields exhibited higher densities of plant-parasitic nematodes compared to other crops and regions. *Paratylenchus* had the highest densities recovered in Saskatchewan's pea and lentil fields, reaching 1,024 per 100 grams of pea soil and 901 per 100 grams of lentil soil. Additionally, Saskatchewan recorded the highest density of *Pratylenchus*, with 630 nematodes per 100 grams of pea soil, and Telotylenchinae, with 980 nematodes per 100 grams in thistle soil from a pea field. In contrast, *Helicotylenchus* had the highest density observed in pea fields in Alberta, with 506 nematodes per 100 grams of soil, also from a pea field.

*Nematode abundance in lentil fields*: Lentil plants hosted a limited number of nematode taxa, and population densities were low. Only four nematode taxa — *Anguina*, *Aphelenchoides, Aphelenchidae* and *Ditylenchus* — were recovered from lentil pods, stems and leaves, all with mean densities of less than one nematode per g^−1^ (Table 5). In soil samples from lentil fields, a high mean population density of 355 nematodes per 100 g^−1^ dry soil was recorded for *Paratylenchus.* Relatively low densities were observed for the *Pratylenchus* and Telotylenchinae groups.

**Table 5:** Mean population densities for plant samples (nematodes per gram) and soil (nematodes per 100 g dry soil mass) positive for taxa of plant-parasitic nematodes from commercial lentil fields in the Canadian Prairies sampled in 2014 and 2015.

**Taxa**	**Above-Ground Crop**					
	**Pods**		**Stems/Leaves**		**Soil Under Crop**	
			
	**Mean**	**Max^a^**	**Mean**	**Max**	**Mean**	**Max**
*Anguina*	-^b^	-	<1	n/a^c^	-	-
*Aphelenchoides*	<1	1	<1	n/a	43	169
Aphelenchidae	<1	1	<1	1	18	41
*Ditylenchus*	<1	n/a	-	-	16	40
*Paratylenchus*	-	-	-	-	355	901
*Pratylenchus*	-	-	-	-	7	n/a
Telotylenchinae	-	-	-	-	23	84

a Max = highest population density observed in a sample.

b – = absence of nematode population of that taxon.

c n/a = not provided because nematode genera was found in a single sample.

*Nematode abundance in chickpea fields*: Only one taxon, Aphelenchidae, was recovered from chickpea plant samples (Table 6). *Ditylenchus and Aphelenchoides* were the only genera recovered from thistle plant samples in chickpea fields. *Ditylenchus* displayed a relatively high density of 125 nematodes per g^−1^ thistle stems and leaves, while Aphelenchidae and *Aphelenchoides* had average densities of less than one nematode per g^−1^ (Table 6). In chickpea soil samples, *Aphelenchoides* had the highest density (36 nematodes per 100 g^−1^ dry soil), followed by *Paratylenchus* (35 nematodes per 100 g^−1^ dry soil) and *Helicotylenchus* (27 nematodes per 100 g^−1^ dry soil).

**Table 6:** Mean population densities for plant samples (nematodes per g) and soil (nematodes per 100 g dry soil mass) positive for taxa of plant-parasitic nematodes from commercial chickpea fields in the Canadian Prairies sampled in 2014 and 2015.

**Taxa**	**Above-Ground Crop**		**Above-Ground Thistle**							
			
	**Stems/Leaves**		**Flowers**		**Stems/Leaves**		**Soil Under Crop**		**Soil Under Thistle**	
					
	**Mean**	**Max^a^**	**Mean**	**Max**	**Mean**	**Max**	**Mean**	**Max**	**Mean**	**Max**
*Aphelenchoides*	-^b^	-	<1	n/a	<1	n/a	36	108	35	n/a
Aphelenchidae	<1	n/a^c^	-	-	-	-	18	25	5	n/a
*Ditylenchus*	-	-	-	-	125	n/a	8	16	-	-
*Helicotylenchus*	-	-	-	-	-	-	27	n/a	-	-
*Hoplolaimus*	-	-	-	-	-	-	4	n/a	-	-
*Paratylenchus*	-	-	-	-	-	-	35	72	-	-
*Pratylenchus*	-	-	-	-	-	-	16	27	90	127
Telotylenchinae	-	-	-	-	-	-	11	23	-	-

a Max = highest population density observed in a sample.

b – = absence of nematode population of that taxon.

c n/a = not provided because nematode genera was found in a single sample.

*Pratylenchus* were more abundant in thistle soil (90 nematodes per 100 g^−1^ dry soil) than in chickpea soil (17 nematodes per 100 g^−1^ dry soil).

*Species identification of main plant-parasitic nematodes by molecular analyses — Ditylenchus spp.: Ditylenchus weischeri* and *D. dipsaci* were identified using species-specific PCR, PCR-RFLP, and sequencing (Table 7). With the exception of one sample, the majority of the analyzed samples from thistle and pea plants and soil were identified as *D. weischeri*. The exception was a pea pod sample from a field in Manitoba that was identified as *D. dipsaci* with all three methods (PCR, PCR-RFLP, and sequencing).

**Table 7:** Species identity by species-specific PCR, RFLP and sequencing of the ITS, 18S and D2-D3 regions of the 28S rDNA gene of *Ditylenchus* individuals recovered from soil and plant sample types.

							**Sequencing**					
**Province**	**Crop**	**Sample Type**	**Field ID**	**No of individual nematodes analyzed**	**Species-specific PCR Identity**	**RFLP Identity**	**Identity**	**Accession**	**Query Cover %**	**Size (bp)**	**Identity %**	**Gene (Primer)**
Manitoba	Pea	Pea pods	7	7	*D. dipsaci*	*D. dipsaci*	*D. dipsaci*	MG384731	100	368	98.00	ITS (TW81-AB28)
Manitoba	Pea	Thistle flowers	8	1	*D. weischeri*	*a*	*D. weischeri*	MG386859	100	676	99.41	ITS (AB28-TW81)
Manitoba	Pea	Thistle flowers	16	8	*D. weischeri*	*D. weischeri*	*D. weischeri*	MG386878	100	457	99.34	ITS (TW81-AB28)
Manitoba	Pea	Thistle flowers	20	1			*D. weischeri*	MG386851	100	471	98.73	ITS (TW81-AB28)
Manitoba	Pea	Thistle stems and leaves	20	1			*D. weischeri*	MG386873	100	555	100	ITS (TW81-AB28)
Manitoba	Pea	Pea pods	23	1	*D. weischeri*							
Manitoba	Pea	Thistle flowers	24	8	*D. weischeri*	*D. weischeri*	*D. weischeri*	MG386859	98–100	676–695	99.70–99.86	ITS (TW81-AB28)
Saskatchewan	Pea	Thistle flowers	25	3	*D. weischeri*							
Saskatchewan	Pea	Thistle stems and leaves	25	1	*D. weischeri*							
Manitoba	Pea	Thistle flowers	28	16	*D. weischeri*	*D. weischeri*						
Alberta	Pea	Pea pods	31	1	*D. weischeri*	*D. weischeri*						
Alberta	Pea	Pea stems and leaves	31	1		*D. weischeri*						
Saskatchewan	Pea	Thistle flowers	39	1	*D. weischeri*							
Saskatchewan	Pea	Thistle stems and leaves	39	3	*D. weischeri*							
Alberta	Pea	Thistle stems and leaves	40	1	*D. weischeri*							
Alberta	Pea	Thistle soil	41	1	*D. weischeri*							
Alberta	Pea	Thistle stems and leaves	44	1			*D. weischeri*	MG386859	99	700	99.57	ITS (TW81-AB28)
Alberta	Pea	Pea soil	57	1	*D. weischeri*							
Alberta	Pea	Thistle soil	57	3	*D. weischeri*							
Alberta	Pea	Thistle flowers	59	5	*D. weischeri*		*D. weischeri*	MG386825	100	680	97.79	ITS (AB28-TW81)
Saskatchewan	Pea	Thistle soil	70	5	*D. weischeri*		*D. weischeri*	MG386878	98	504	100	ITS (ITSF-ITSR)
Alberta	Chickpea	Thistle stems and leaves	76	8	*D. weischeri*		*D. weischeri*	MG386825 MG386869	99–100	642–679	98.8–99.00	ITS (AB28-TW81)
Saskatchewan	Pea	Thistle stems and leaves	148	2	*D. weischeri*							
Alberta	Pea	Thistle soil	165	3	*D. weischeri*							
Saskatchewan	Pea	Thistle stems and leaves	186	5	*D. weischeri*		*D. weischeri*	MG551907	99	694	99.85	28S (D2A-D3B)
Saskatchewan	Pea	Thistle stems and leaves	190	1	*D. weischeri*							

a blank cells indicate no analysis conducted.

A few samples were not successfully assigned to species. No amplification was detected for 30 DNA samples from six fields with *Ditylenchus weischeri* and *D. dipsaci* species-specific PCR. Additionally, eight *Ditylenchus* sequences from eight fields had low identity values or query cover, and thus could not be assigned to species. As a result, *Ditylenchus* from 14 fields could not be identified to species, possibly due to the presence of unknown species and limitations in the analysis process.

*Pratylenchus spp.*: PCR analysis and sequencing results revealed the presence of *P. neglectus* in multiple pea, chickpea, and thistle soil samples across the studied fields. Other *Pratylenchus* species, such as *P. penetrans*, *P. thornei*, and *Pratylenchus scribineri* Steiner, 1943, were not detected in the samples analyzed. PCR with *P. neglectus* species-specific primers yielded a single positive 290-bp band for 16 out of the 35 specimens tested (Table 8). The same 35 specimens were also tested with *Pratylenchus penetrans* (Cobb) Filipjev & Schuurmans-Stekhoven and *P. thornei* species-specific primers, yielding no amplification. *Pratylenchus penetrans*, *P. thornei* and *P. scribineri* primers were also tested in 25, 37, and 11 *Pratylenchus* DNA samples, respectively, representing eight fields. Results either failed to produce a band or produced bands of the wrong size. However, we caution that the absence of positive controls in these reactions limits our ability to interpret the results and draw definitive conclusions about the presence or absence of these species. Sequencing of the 18S and the D2–D3 regions of ribosomal DNA revealed that 17 specimens from five fields had the highest similarity with *P. neglectus* (Table 8). However, two *Pratylenchus* sequences from one field had only a 96% identity match with *P. neglectus*, and were classified as unknown species because they did not meet the cut-off criteria for species assignment (≥ 98% identity score).

**Table 8:** Species characterization by species-specific PCR and sequencing of the ITS, 18S and D2-3 regions of the 28S rDNA gene of *Pratylenchus* individuals recovered from soil samples.

						**Sequencing**					
**Province**	**Crop**	**Sample Type**	**Field ID**	**No of individual nematodes analyzed**	**Species-specific PCR Identity**	**Identity**	**Accession**	**Query Cover %**	**Size (bp)**	**Identity %**	**Primer**
Alberta	Pea	Pea soil	98	1	*^a^*	*P. neglectus*	KY424172	100	547	99.82	18S (18SF-18SR)
Alberta	Pea	Pea soil	131	1		*P. neglectus*	JQ303332	100	865	99.65	18S (18SF-18SR)
Alberta	Pea	Thistle soil	50	7	*P. neglectus*	*P. neglectus*	MT261132	100	758	99.08	28S (D2A-D3B)
Alberta	Chickpea	Chickpea soil	69	1	*P. neglectus*						
Alberta	Chickpea	Thistle soil	90	3	*P. neglectus*						
Saskatchewan	Pea	Pea soil	185	185-1	unknown	*P. neglectus*	JQ303332	99	823	99.76	18S (18SF-18SR)
Saskatchewan	Pea	Pea soil	185	185-4	*P. neglectus*						
Saskatchewan	Pea	Pea soil	185	185-5	*P. neglectus*						
Saskatchewan	Pea	Pea soil	185	185-6	*P. neglectus*						
Saskatchewan	Pea	Thistle soil	184	184-2	unknown	*P. neglectus*	KM200579	100	667	99.40	28S (D2A-D3B)
Saskatchewan	Pea	Thistle soil	184	184-5	*P. neglectus*						
Saskatchewan	Pea	Thistle soil	192	192-1	*P. neglectus*						

a blank cells indicate no analysis conducted.

*Paratylenchus spp.: Paratylenchus* field populations were identified through sequencing of the ITS region of the 18S rDNA and the D2–D3 expansion segments of the 28S ribosomal subunit as *P. projectus* (Table 9). Positive samples for *P. projectus* were obtained from one pea field in Alberta, one pea field in Saskatchewan, and two lentil fields in Saskatchewan.

**Table 9:** Sequencing of the ITS, 18S and the D2–D3 region of the 28S rDNA gene of *Paratylenchus* individuals recovered from soil samples.

					**Sequencing**					
**Province**	**Crop**	**Sample Type**	**Field ID**	**No of individual nematodes analyzed**	**Identity**	**Accession**	**Query Cover %**	**Size (bp)**	**Identity %**	**Primer**
Alberta	Pea	Pea soil	33	1	*P. projectus*	MW413605	97	567	99.8	ITS (ITSF-ITSR)
Saskatchewan	Pea	Pea soil	62	2	*P. projectus*	MW413656	99–100	646–708	99.69–100	28S (D2A-D3B)
Saskatchewan	Lentil	Lentil soil	120	1	*P. projectus*	MW413605	99	579	99.65	ITS (ITSF-ITSR)
Saskatchewan	Lentil	Lentil soil	130	1	*P. projectus*	KJ636434	100	852	99.77	18S (18SF-18SR)
Saskatchewan	Lentil	Lentil soil	130	1	*P. projectus*	MW413605	97	584	97.36	ITS (ITSF-ITSR)

*Aphelenchoides spp*.: A few *Aphelenchoides* nematodes suspected of being plant-parasitic were tested using species-specific PCR and sequencing. Nematodes were selected for DNA extraction based on morphological features, such as lip and caudal region. Results from the species-specific PCR analyses did not confirm the presence of *A. besseyi*, *A. ritzemabosi*, *A. fragariae*, or *A. subtenuis* among the tested *Aphelenchoides* nematodes; sequencing results also did not provide sufficient information to identify the specimens at the species level.

DNA samples from 22 *Aphelenchoides* nematodes from two pea fields (pea and thistle plant samples in Alberta) were tested with *A. besseyi*, *A. ritzemabosi, A. fragariae* and *A. subtenuis* Cobb species-specific primers. The results showed no specific amplification, but we caution that the absence of positive controls in these reactions means we cannot conclude with certainty that those species were not present in the fields tested. Further analysis involved sequencing nine specimens from lentil and pea plant samples from five fields in Alberta and Saskatchewan. The results showed a low identification match and query cover, and therefore specimens could not be assigned at species level.

*Quinisulcius spp*.: DNA sequences of the partial ITS, 18S rRNA gene, and the D2–D3 expansion segments of the 28S rDNA were obtained for 15 specimens, and they closely matched the species *Quinisulcius capitatus* (Allen 1955) Siddiqi, 1971. The positive samples were from three pea fields in Alberta (pea soil sample) and one in Saskatchewan (thistle soil sample).

*Other genera*: Other species identified through sequencing were *Merlinius brevidens* (Allen, 1955) Siddiqi, 1970 (*Geocenamus brevidens* (Allen, 1955) Brzeski, 1991) (one field, pea soil, AB), and *Aphelenchus avenae* Bastian, 1865 (two fields, pea soil and pea stems and leaves, AB).

Two *Xiphinema* nematodes recovered from Alberta's pea soil samples had the highest similarity to *Xiphinema rivesi* Dalmasso, 1969, based on sequencing of the D2–D3 region of the 28S rDNA gene.

A BLAST search of the D2–D3 region of the 28S rDNA and the ITS region of the rDNA could not confirm genus- or species-level identities for one *Pratylenchus*, one *Helicotylenchus*, four *Paratylenchus*, three *Tylenchorhynchus*, and six unidentified genera.

## Discussion

Our findings confirm the widespread presence of *D. weischeri* in commercial pulse crop fields across the Canadian Prairies and indicate a limited prevalence of *D. dipsaci*. This agrees with the findings of Tenuta et al. (2014), which suggested that *D. weischeri* is the predominant species present in thistles, contesting an earlier report by Watson and Shorthouse (1979) regarding *D. dipsaci* infestation in thistles.

In our study, *D. dipsaci* was found at a low density exclusively in pea pods within a single yellow pea field in Rhineland, Manitoba, while no presence was detected in leaves and stems or soil samples. We re-sampled the same field, plus adjacent ones the following year (in 2016), and did not recover *D. dipsaci* from the soil (unpublished data). These fields had canola and soybean in rotation that year; therefore, no plant samples were collected.

In the second year of the survey — the same year we found *D. dipsaci* in Rhineland — significant economic losses caused by *D. dipsaci* were reported in two garlic fields in southern Manitoba (Hajihassani and Tenuta, 2017). The grower had obtained the contaminated garlic seed pieces from Ontario, which was known for having *D. dipsaci*. A similar situation was subsequently reported in Alberta garlic fields in 2020 (Harding et al. 2021). Investigations by Hajihassani et al. (2016) revealed that *D. weischeri* does not parasitize chickpeas or lentils, unlike *D. dipsaci*, which has been reported to infest lentils and weakly reproduce in chickpeas (Greco and Di Vito, 1994). Greenhouse studies have shown that *D. weischeri* exhibits weak reproduction in two pea varieties, but requires an average temperature of 27ºC for complete development and reproduction, which is atypical and unsustainable in the Canadian Prairies (Hajihassani et al., 2017).

Canadian yellow pea exports have been unaffected by the presence of *D. dipsaci* in a single pea field in Manitoba, as the province does not export field pea grain. However, it is concerning that *D. dipsaci* was also found in garlic fields elsewhere in Manitoba, since it has a wide range of suitable host crops and potential to infect other crops grown within the vicinity of infested garlic fields (Hajihassani and Tenuta, 2017). Measures to prevent transmission, such as implementing strict biosecurity protocols, should be taken, since this nematode is challenging to control once established in high numbers.

*Pratylenchus neglectus* and other *Pratylenchus* species have been identified in previous studies in the Canadian Prairies (Mahran et al., 2010; Yu, 2008). We identified *P. neglectus* in crop and thistle soil samples from pea fields in Saskatchewan and Alberta, and in chickpea soil in Alberta, but did not identify any other *Pratylenchus* species. The number of samples we tested was not comprehensive and did not represent all the fields surveyed; it is likely that other species are present.

The economic importance of *P. neglectus* in the Canadian Prairies is poorly understood, but the species has been associated with potatoes and wheat in Alberta (Forge et al., 2015) and is known as a major parasite of cereals in the United States (Smiley et al., 2005). Significant economic damage — up to 90% production loss — has been reported in Idaho's dryland pea and lentil crops due to mixed populations of *P. neglectus*, *P. thornei*, and *Paratylenchus hamatus* (Riga et al., 2008).

Wenyika (2019) conducted a host preference study using soils naturally infested with *P. neglectus* that had been obtained from a field in Alberta; the same field was also surveyed in the present study. The results revealed that most pulse and non-pulse crops grown in the Canadian Prairies — specifically, chickpea, canola, soybean, pinto bean and spring wheat — are suitable hosts for this nematode species. However, lentil was a poor host and yellow pea was a nonhost for *P. neglectus* (Wenyika, 2019).

For pulse crops — namely, beans and cowpeas — a threshold of 50 and 100 nematodes per 100 cm^−3^ of soil has been reported for *Pratylenchus* for different soil types (Dickerson et al. 2000). In general, a threshold of 100 nematodes per 100 mL^−1^ of soil has been established for *Pratylenchus* spp. for most crops (Rivoal and Cook, 1993; Thompson et al., 2010; Fleming et al., 2016). In this survey, seven out of 19 fields positive for *Pratylenchus* exceeded 100 nematodes per 100 g^−1^ of soil. Six of the highly infested fields were positive for *P. neglectus*, indicating a potential concern for farmers. The highest density of *P. neglectus* (630 nematodes per 100 g^−1^ dry soil) was observed in a pea soil sample from a field in Saskatchewan, while thistle soil from this field contained only 158 nematodes per 100 g^−1^ dry soil. This finding is consistent with studies conducted in the United States and Bulgaria, which indicate that thistle is a poor or non-host for this nematode species (Samaliev and Markova, 2014; Smiley et al., 2014). Pea crops are typically grown in rotation with soybean, canola, and wheat, all of which have been recognized as favorable hosts for *P. neglectus* (May et al., 2016; Wenyika, 2019). Previous research has indicated that populations of *Pratylenchus* can experience substantial growth when cereals are frequently cultivated in the rotation cycle (Smiley et al., 2005). Consequently, *P. neglectus* populations found in this survey might be subsisting on peas and thistles, and may increase when a more suitable host is added to the rotation.

*Paratylenchus*, commonly known as pin nematodes, had the highest mean density among the nematode genera recovered from lentil and pea crops in this survey. *Paratylenchus* threshold limits have not been established for pulse crops, but it has been reported that grasses and cereals have a threshold range of 51 to 300 nematodes per 100 g^−1^ soil (Dickerson et al., 2000). This threshold was exceeded by most of the lentil and pea fields positive for *Paratylenchus* in our survey. Notably, the fields in Saskatchewan had the highest densities, exceeding 300 *Paratylenchus* per 100 g^−1^ of soil.

Chickpea fields had low mean population densities of *Paratylenchus*, with only two fields, both in Saskatchewan, surpassing the lower threshold. However, since only three chickpea fields in Saskatchewan were sampled, results drawn from this subset of fields cannot fully represent the overall population densities associated with *Paratylenchus* in chickpea fields.

Using sequencing methods, we identified only one *Paratylenchus* species: *P. projectus*. *Paratylenchus projectus* is considered an important pest of forage (Ghaderi, 2019), legumes and grasses (Townshend and Potter, 1976; Ghaderi, 2019). This species was recovered from the two fields with the highest *Paratylenchus* densities in this survey, with 901 nematodes per 100 g^−1^ dry pea soil and 1,024 nematodes per 100 g^−1^ dry lentil soil in Saskatchewan. *Paratylenchus projectus* have been previously reported in Alberta (Hawn, 1973; Webster and Hawn, 1973) and Ontario (Senwel, 1971) pea fields. To our knowledge, this is the first report of *P. projectus* in Saskatchewan.

Several taxa of stunt nematodes (Dolichodoridae) were identified, including members of the subfamily Telotylenchinae (*Tylenchorhynchus* and *Quininsulcius capitatus*) and the subfamily Merliniinae (*Merlinius brevidens*).

*Tylenchorhynchus* is a pest that affects chickpeas (Maqbool, 1987) and wheat. In a wheat field survey in Montana, USA, *Tylenchorhynchus* were widely distributed and had high population levels, indicating that this nematode may be of concern to regional wheat producers (Johnson, 2007). A guideline report from South Carolina, USA (Dickerson et al., 2000) suggested the following thresholds for *Tylenchorhynchus*: 100 nematodes per 100 cm³ of soil for wheat; 200 to 300 nematodes per 100 cm³ of soil for beans; and more than 500 nematodes per 100 cm³ of soil for corn and soybean crops. Our survey found that 15 fields exceeded the wheat threshold, and some even surpassed the highest threshold limits recommended for soybean and corn by Dickerson et al. in 2000. These findings are concerning because wheat, corn, and soybean are often grown in rotation with peas, and thus may be vulnerable to the high density of *Tylenchorhynchus* found during this survey.

We identified *Quinisulcius capitatus* from three fields in Alberta and one in Saskatchewan. The positive samples from Alberta, obtained from pea soil, showed densities ranging from 30 to 123 nematodes per 100 g of dry soil. In contrast, the sample from Saskatchewan, collected from thistle soil, had a density of 26 nematodes per 100 g of dry soil. *Quinisulcius capitatus* is a newly identified species in Canada that was recently described in potato-growing regions in Alberta (Munawar, 2021). In other countries, it has been associated with pea and soybean (Bridge, 1976; Mbatyoti et al., 2020), potato (Hafez et al., 2010), and other crops (Bridge, 1976).

Merliniinae (stunt nematode) were found in pea soil samples in very low numbers in this survey. *Merlinius brevidens* was recovered from one pea field in Alberta at a density of 10 nematodes per 100 g^−1^ dry soil, and was the only species identified through sequencing. *Merlinius brevidens* was reported in Alberta (Munawar et al., 2021) and has been associated with yellow pea (Pinkerton et al., 1999) and chickpea (Castillo et al., 2008). However, the low density and frequency of *Merlinius* in this survey were not high enough for the genus to be considered a concern for growers.

*Helicotylenchus* spp. have an extensive host range, including chickpeas (Askary, 2017), peas (Wouts and Knight, 1993) and lentils (Marais and Swart, 1996). Out of the 25 fields that were positive for *Helicotylenchus*, seven had nematode population densities above 100 nematodes per 100 g^−1^ dry soil (all of which were from either pea soil or thistle soil samples in pea fields), with a maximum density of 506 *Helicotylenchus* per 100 g^−1^ dry soil. While densities of 100 nematodes per 100 cm^−3^ were enough to cause damage to corn and soybean in Iowa, USA (Norton and Nyvall, 1999), other researchers have suggested that higher densities of this nematode are necessary to damage crops (Mekete et al., 2011; Niblack and Paul, 2014).

*Aphelenchoides* (Aphelenchoididae) were frequently found in this survey, but population densities were relatively low. Most *Aphelenchoides* spp. are primarily mycophagous and found in soil (Ruess et al., 2000; Duncan and Moens, 2013). Only a few *Aphelenchoides* species pose a significant threat to crops, such as *A. bessey*, *A. fragariae* and *A. ritzemabosi* (Duncan and Moens, 2013). We were unable to identify any *Aphelenchoides* species; our sequencing results did not yield a close match to any in the BLAST database, and our attempts to use species-specific PCR with *A. besseyi*, *A. ritzemabosi*, *A. fragariae*, and *A. subtenuis* primers failed to produce the expected band size. These findings indicate an absence of *Aphelenchoides* plant-parasitic nematodes in the fields we tested.

Aphelenchidae were recovered at low population densities for all crops and sample types in this survey. *Aphelenchus avenae* was identified through sequencing from pea soil and pea stem and leaf samples from two fields in Alberta. *Aphelenchus avenae* is primarily a fungal feeder; it can be a higher plant parasite, although reports of the pathogenicity of this nematode are few (Barker and Darling, 1965; Kumari, 2012). It was previously reported in samples of native grasses in Saskatchewan, as well as alfalfa in Alberta (Sewell, 1977).

*Xiphinema* was not prominent in the fields surveyed in this study. This is consistent with a survey taken in Minnesota's northwestern region (Chen et al., 2012). Two specimens recovered from Alberta pea soil samples showed high similarity to *X. rivesi*, which is not a parasite of pulse crops.

Other important genera of nematodes isolated from soil samples in this study include *Longidorus*, *Paratrichodorus*, and *Hoplolaimus*; however, the population densities and frequency of these nematodes were low. Although some species of these genera are of economic significance among major crops (Nicol et al., 2011), the low frequency and density values found in this survey suggest that they are not a significant concern for pulse growers in the Canadian Prairies at this time.

It is important to note that the primary focus of our survey was *Ditylenchus*, and this influenced sampling methods and field selection. Since *Ditylenchus* is a foliar nematode, we analyzed above-ground vegetation and not roots, which could have provided valuable insights into the association between root-feeding nematodes, such as *Pratylenchus*, and pulse crops.

Moreover, the soil extraction method employed favored certain nematode groups while missing others, such as Heteroderidae. Although the Soil Ecology Lab at the University of Manitoba is conducting ongoing cyst nematode surveys to address Heteroderidae, other groups remain underrepresented, as the primary objective of this study was on *Ditylenchus* distribution. Field selection was also strongly influenced by *Ditylenchus dipsaci*, a major pest of yellow peas, which led us to prioritize pea fields. Consequently, lentil and chickpea fields were underrepresented, potentially missing nematode species with a strong preference for those crops. Although we collected and analyzed 466 samples, a simplified approach to combining above-ground plant samples could have allowed for sampling additional chickpea and lentil fields. Nevertheless, we achieved our research objectives, and our survey highlights the importance of examining foliar nematodes.

In conclusion, *D. dipsaci* is nearly absent in pea fields in the Prairie provinces, with only a single detection in a pea field in Manitoba, while *D. weischeri* is prevalent in thistles and is not associated with major crops commonly grown in the Canadian Prairies. *Pratylenchus, Paratylenchus*, Telotylenchinae and *Helicotylenchus* were found in soil samples at densities above suggested threshold levels in some fields, raising potential concerns for growers*. Pratylenchus neglectus* was present in most samples above its suggested threshold limits. Other species of concern found in this survey were *Quinisulcius capitatus, Paratylenchus projectus, Merlinius brevidens, Xiphinema rivesi and Aphelenchus avenae*. The data presented in this study adds to our understanding of which plant-parasitic nematodes pose existing or potential problems for pulse crops in the Canadian Prairies. Further studies to monitor *Ditylenchus dipsaci* and *Pratylenchus* population dynamics are warranted.
